# Accelerated atherosclerosis in HGPS

**DOI:** 10.18632/aging.101608

**Published:** 2018-10-21

**Authors:** Magda R. Hamczyk, Vicente Andrés

**Affiliations:** 1 Centro Nacional de Investigaciones Cardiovasculares (CNIC), Madrid, Spain.

**Keywords:** aging, HGPS, progerin, atherosclerosis, vascular smooth muscle cell

Aging is the principal risk factor for cardiovascular disease (CVD), the leading cause of death worldwide. The yearly death toll from CVD is 17.7 million people, representing 31% of all deaths (www.who.int/cardiovascular_diseases). Most clinical manifestations of CVD (e.g. myocardial infarction and stroke) derive from atherosclerosis, which is characterized by thickening of the arterial wall and luminal narrowing due to atherosclerotic plaque accumulation. Accelerated atherosclerosis is a key feature of Hutchinson-Gilford progeria syndrome (HGPS), an ultrarare genetic disorder caused by a *de novo* heterozygous point mutation (c.1824C>T; p.G608G) in the *LMNA* gene, resulting in the expression of a toxic form of lamin A called progerin [1]. Affected children prematurely manifest features of aging, such as skin abnormalities, alopecia, lipodystrophy, joint stiffness, and reduced bone density. Although most HGPS patients lack classical cardiovascular risk factors, they typically die from atherosclerosis complications at an average age of 14.6 years. Thus, HGPS gives us an exceptional opportunity to study CVD independently of classical risk factors such as elevated low-density cholesterol and tobacco smoking [2].

To facilitate research into HGPS, several mouse models have been developed that resemble many features of the human disease [2]. However, none of the available models develops atherosclerosis, the death-causing symptom of this devastating disease. To bridge this gap, we generated an atherosclerosis-prone HGPS-like mouse model [3] by crossing progeroid *Lmna*^G609G^ knock-in mice [4] with atherosclerotic *Apoe* knockout mice. Similar to HGPS patients, progeroid *Apoe^-/-^Lmna*^G609G/G609G^ mice have a severe premature aging phenotype with impaired postnatal growth and a shortened lifespan (median survival 4.5 months, compared with 29.4 months for control mice). Importantly, *Apoe^-/-^Lmna*^G609G/G609G^ mice have a higher atherosclerosis burden than control *Apoe^-/-^* mice with a wild-type *Lmna* gene, despite having similar serum cholesterols levels. *Apoe^-/-^Lmna*^G609G/G609G^ mice also develop other vascular pathologies previously detected in postmortem specimens from HGPS patients [5], including vascular smooth muscle cell (VSMC) loss in the media and adventitial thickening. We also found lipid deposits in the medial aorta of these mice, a phenomenon not described before. All these vascular alterations are age- and high-fat diet-dependent, suggesting that progerin-expressing tissues have an exacerbated response to aging and high cholesterol.

Atherosclerosis involves many cell types, including leukocytes and cells of the arterial vessel wall. To identify the main cell type(s) responsible for the progerin-driven acceleration in atherogenesis, we generated mouse models with cell type-specific progerin expression. We focused on VSMCs and macrophages, since findings from progeria mice and HGPS patient samples have implicated these cells types as potential contributors to atherosclerosis in HGPS [3,5]. Macrophage- and VSMC-specific progerin-expressing mice show no overt aging phenotype and are indistinguishable from controls until approximately 5 months of age, when VSMC-specific mice stop gaining weight and die at an average age of 8.6 months (median survival for control mice is 26.6 months and for the macrophage-specific model 29 months). VSMC-specific mice, but not macrophage-specific mice, present adventitial thickening, medial VSMC depletion and lipid deposits, and enhanced atherosclerosis, fully resembling the vascular phenotype of the ubiquitous progeria mice.

VSMC death during atherosclerosis has been linked to the vulnerable plaque phenotype [6], and we therefore investigated the consequences of progerin-induced VSMC loss on plaque stability and composition. Atheromas in both the ubiquitous and the VSMC-specific progeria mice had bigger necrotic cores, loss and disorganization of VSMCs in the fibrous cap, and iron deposits, indicating plaque instability that can lead to myocardial infarction.

The exacerbated atherosclerosis in *Apoe^-/-^* mice with ubiquitous and VSMC-specific progerin expression occurs in the presence of serum cholesterol levels similar to those in *Apoe^-/-^* controls. However, both progeria models exhibit abnormally high medial lipid accumulation, coinciding with VSMC loss and replacement by extracellular matrix. Experiments with fluorescently-labeled human low-density lipoproteins revealed more lipoprotein retention in the aortic wall in both the ubiquitous and the VSMC-specific progeria mice, which may contribute to the enhanced atheroma plaque formation [3]. Further research is warranted to identify additional mechanisms underlying progerin-induced lipid accumulation in the aortic media, such as enhanced endothelial permeability.

**Figure 1:**
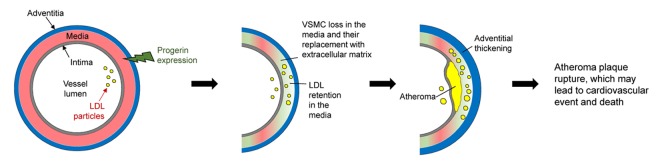
**Proposed model of accelerated atherosclerosis induced by progerin expression in vascular smooth muscle cells (VSMCs).** The aortic wall has three layers: the adventitia, media (containing VSMCs), and intima (containing endothelial cells). Progerin expression in VSMCs leads to their progressive loss in the aortic media and their replacement by extracellular matrix. These changes, together with other factors, increase low-density lipoprotein (LDL) retention in the aortic wall, which exacerbates atheroma plaque formation. In parallel, the adventitia becomes thicker and denser. Depletion of VSMCs in the atheroma may cause plaque instability and rupture, which ultimately provokes ischemic cardiovascular events (e.g. myocardial infarction) and premature death.

These mouse models, together with findings in tissues from HGPS patients, show that VSMC death plays a central role in premature atherosclerosis in HGPS (Figure 1). However, the molecular mechanisms by which progerin causes VSMC loss in the vessel wall remain to be elucidated. HGPS research also has the potential to shed light on other conditions featuring premature CVD, such as the accelerated atherosclerosis in protease inhibitor-treated HIV patients, whose cells accumulate another toxic form of lamin A (called prelamin A) as a side effect of the treatment. Findings from HGPS studies may also aid understanding of normal aging, since low levels of progerin are found in cells and tissues (including VSMCs) of elderly people [7].
